# Hemodynamic effects of intraoperative anesthetics administration in photothrombotic stroke model: a study using laser speckle imaging

**DOI:** 10.1186/s12868-016-0327-y

**Published:** 2017-01-05

**Authors:** Hongyang Lu, Yao Li, Bin Bo, Lu Yuan, Xiaodan Lu, Hangdao Li, Shanbao Tong

**Affiliations:** 1 School of Biomedical Engineering, Shanghai Jiao Tong University, 800 Dongchuan Road, Shanghai, 200240 China; 2 Med-X Research Institute, Shanghai Jiao Tong University, 1954 Huashan Road, Shanghai, 200030 China

**Keywords:** Laser speckle imaging, Photothrombotic stroke model, Hemodynamic effect, Anesthetics

## Abstract

**Background:**

Previous neuroimaging studies have shown the hemodynamic effect of either preconditioning or postconditioning anesthesia in ischemic stroke model. However, the anesthetic effect in hemodynamics during and immediately after the stroke modeling surgery remains unknown due to the lack of appropriate anesthesia-free stroke model and intraoperative imaging technology. In the present study, we utilized our recently developed photothrombotic model of focal cerebral ischemia in conscious and freely moving rats, and investigated transient hemodynamic changes with or without isoflurane administration. Laser speckle imaging was applied to acquire real-time two-dimensional full-field cerebral blood flow (CBF) information throughout the surgical operations and early after.

**Results:**

Significantly larger CBF reduction area was observed in conscious rats from 8 min immediately after the onset of stroke modeling, compared with anesthetized rats. Stroke rats without isoflurane administration also showed larger lesion volume identified by magnetic resonance imaging 3 h post occlusion (58.9%), higher neurological severity score 24 h post occlusion (28.3%), and larger infarct volume from 2,3,5-triphenyltetrazolium chloride staining 24 h post occlusion (46.9%).

**Conclusions:**

Our results demonstrated that the hemodynamic features were affected by anesthetics at as early as during the stroke induction. Also, our findings about the neuroprotection of intraoperative anesthetics administration bring additional insights into understanding the translational difficulty in stroke research.

## Background

Stroke is the leading cause of disability and mortality worldwide, which occurs when a cerebral vessel is either blocked or hemorrhagic. Ischemic stroke, accounting for more than 80% of all stroke cases, initiates a sequential metabolic and biochemical disorders and subsequently leads to the neuronal apoptosis and necrosis [1]. So far, almost all the laboratorial and pre-clinical stroke studies are based on animal models, in which anesthesia is administered for the concern of animal care and ethics [2–4].

There have been a variety of studies showing the global effect of anesthetics administration in animal stroke model including alteration of neuronal and vascular functions. Inhaled anesthetics such as isoflurane can modulate synaptic transmission and neuronal excitability [5], augment GABA neurotransmission [6], and regulate cerebral blood flow (CBF) [7, 8]. The CBF reduction serves as the most important indicator in ischemic stroke, which is closely related to the volume of brain infarction [9]. Previous neuroimaging studies have shown that the administration of isoflurane in either preconditioning [10] or post-conditioning following ischemia/reperfusion [11] altered the regional hemodynamic variations. The constrained deleterious CBF changes lead to neuroprotective effects such as decrease of brain infarction volume and reduction of intracerebral hemorrhage [12]. However, the hemodynamic observations provided by magnetic resonance imaging (MRI) were constrained to a few time points with insufficient temporal resolution, while the CBF information obtained by laser Doppler flowmetry (LDF) was confined at very limited cerebral locations with poor spatial resolution [13]. Furthermore, due to the lack of appropriate anesthesia-free stroke model, the intraoperative anesthetic effect during and immediately after the stroke modeling surgery remains unknown.

In our recent work [14] and others’ [15], a photothrombotic model of focal cerebral ischemia was induced in conscious and freely moving rats without introducing noticeable pain or stress to the animals. We utilized this model to investigate transient hemodynamic changes with or without isoflurane administration during the photothrombotic ischemic stroke modeling surgery. Laser speckle imaging (LSI) was applied to acquire real-time two-dimensional full-field CBF information throughout the surgical operations and early after. In addition, we measured the brain lesion by MRI 3 h post occlusion, neurological severity score (NSS) and brain infarct volume 24 h post stroke to investigate the potential neuroprotective effects of intraoperative anesthetics administered during stroke.

## Methods

The experimental protocols in this study were approved by the Animal Care and Use Committee of Med-X Research Institute, Shanghai Jiao Tong University.

### Animal preparation

Twenty-two male Sprague–Dawley rats (320 ± 20 g, 12 weeks of age, Slac Laboratory Animal, Shanghai, China) were used in this study. The rats were housed within a research animal facility under a 12-h reverse light/dark cycle in a comfortable environment (temperature: 21–25 °C; humidity: 20–50%) with free access to food and water. A cranial window was prepared 24 h before stroke modeling. During the window preparation, the rat was anesthetized with isoflurane (5% initial and 1.0–1.5% for maintenance) and the rectal temperature was maintained at 37.0 ± 0.2 °C using a heating pad with a control module (FHC Inc., Bowdoin, ME). After a midline incision was made over the scalp, the tissues were cleaned with a scalpel to expose the skull. A 5.0 mm × 7.0 mm window over the left hemisphere, centered at 3.5 mm posterior to the bregma and 2.5 mm lateral to the middle line, was thinned by a high-speed dental drill (Fine Science Tools, Inc., Foster City, CA) until the cortical vessels were clearly visible. A cylinder base (lab-designed, height: 4.2 mm, radius: 5.5 mm, thickness: 0.5 mm) enclosing the thinned area was fixed onto the skull with reinforced glass ionomer cements (Dental Materials Factory of Shanghai Medical Instruments Co., Shanghai, China) to form an imaging chamber. All procedures were performed under standard sterile precautions. After the cement hardened, the animals were caged and provided with sufficient food and water for 24 h to eliminate the effects of isoflurane.

### Photothrombotic stroke modeling

**Fig. 1:**
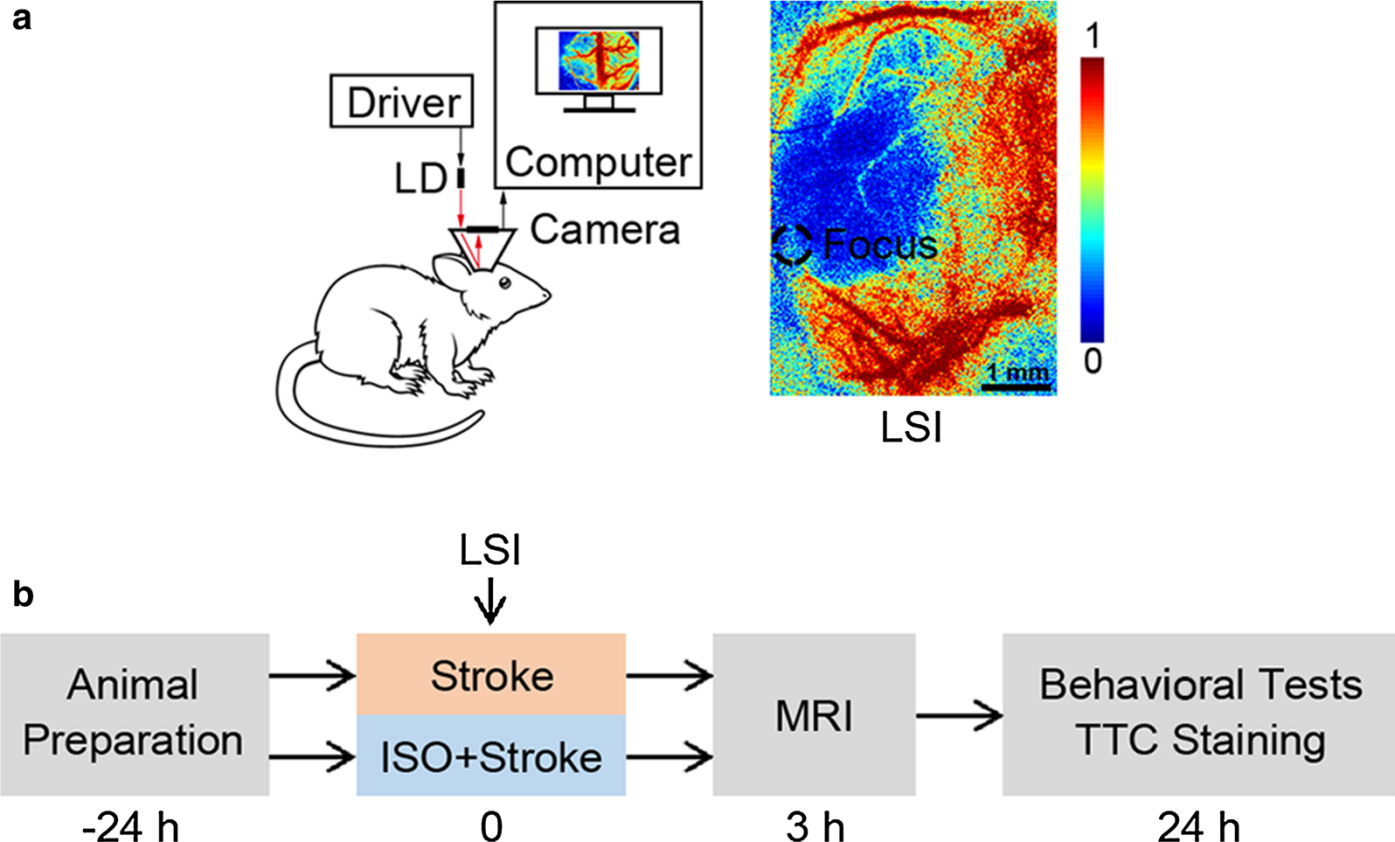
Photothrombotic model of focal ischemic stroke. **a** Schematic of the design of laser speckle imaging system (*left panel*) and a typical LSI image representing CBF information in pseudo-color. *Dashed circle* indicates the focus of the 532 nm light illumination applied in modeling of focal ischemia. *LD* laser diode, *LSI* laser speckle imaging. *Scale bar* 1 mm. **b** Schematic of the experimental protocols. *ISO* isoflurane, *MRI* magnetic resonance imaging, *TTC* 2,3,5-triphenyltetrazolium chloride

The rats were randomly assigned to either conscious (*n* = *11*) or anesthetic group (*n* = *11*). The photothrombotic stroke modeling procedure has been detailed in our previous work [14]. In short, after caged for 24 h, all rats were constrained briefly in order to connect the headstage to the cylinder base for stroke modeling and CBF imaging. Rose Bengal (Sigma-Aldrich Co. LLC., St. Louis, MO) was injected intravenously (80 mg/kg body weight) into tail veins. A 532 nm laser beam (focus diameter: ~750 μm, power: 5 mW) was directed and focused at the Y-shaped juncture of the parietal branches of distal middle cerebral artery (MCA) [16], by a single-mode optical fiber (modified from P1-460B-FC-1, Thorlabs, Newton, NJ) along with an aspheric lens. The fiber was rigidly fixed onto the supporting frame of the headstage to avoid displacement. The focus of illumination could be adjusted by tuning the anchor screws so that the ischemic core was consistently selected in all animals (Fig. 1a). Ischemia was induced through photoactivation of the pre-injected photosensitizing dye (i.e. Rose Bengal), which consequentially resulted in platelet aggregation and vascular thrombosis with the illumination of the laser beam, as described in previous literature [17, 18]. In this study, thrombosis could be created within 15 min of illumination (Fig. 1a). To minimize the potential influence of motion activities, each rat in the conscious group was constrained in a small rearing cage during the experiment. For the anesthetic group, the procedures were identical to those in the conscious group, except that the rats were anesthetized with isoflurane (5% initial and 1.0–1.5% for maintenance) starting from 30 min prior to stroke modeling till the end of illumination. The schematic of the experimental protocol is illustrated in Fig. 1b.

### Real-time cerebral blood flow monitoring

Since we focused on the intraoperative anesthetic influence on CBF, only up to 25 min post-occlusion CBF data were recorded. Raw laser speckle images (640 × 640 pixels) were continuously recorded at 50 fps after the headstage was connected to the cylinder base. The speckle contrast *K*_*s*_ is theoretically linked to the blood flow velocity by

1 $$ K_{s}^{2} = \frac{{\sigma_{s}^{2} }}{{\left\langle I \right\rangle^{2} }} = \beta \left\{ {\frac{{\tau_{c} }}{T} + \frac{{\tau_{c}^{2} }}{{2T^{2} }}\left[ {\exp \left( { - \frac{2T}{{\tau_{c} }}} \right) - 1} \right]} \right\} $$

where *T* is the exposure time of the CCD camera and the autocorrelation time *τ*_*c*_ is assumed to be inversely and linearly proportional to the mean speed of the blood flow [19]. *β* accounts for the loss of correlation, which is related to the ratio of the detector size to the speckle size and polarization [20]. All image processing algorithms were implemented in MATLAB^®^ (Mathworks, Natick, MA). After removing the motion artifacts using image registration algorithm [21], the random process estimator was applied to obtain the CBF information [22]. Regional CBF changes in distal MCA were monitored to confirm the success of stroke modeling. We normalized all CBF images after ischemia by the baseline before the stroke, pixel by pixel so as to obtain relative CBF information,

2 $$ N_{{\left( {x,y} \right)}} \left( t \right) = \frac{{{\text{CBF}}_{{\left( {x,y} \right)}} \left( t \right)}}{{{\text{CBF}}_{{\left( {x,y} \right)}} \left( 0 \right)}} $$

where *N*_(*x*,*y*)_(*t*) is the normalized blood flow velocity at pixel (*x*,*y*).

Relative CBF (rCBF) changes in distal MCA were detected and calculated. Moreover, we computed the affected area in both groups by selecting the pixels in the ipsilateral hemisphere that represented an over 50% reduction compared with baseline value prior to stroke modeling [9],

3 $$ B_{{\left( {x,y} \right)}} \left( t \right) = \left\{ {\begin{array}{*{20}c} {1,} &\quad {N_{{\left( {x,y} \right)}} \left( t \right) < 50\% } \\ {0,} &\quad {\text{otherwise}} \\ \end{array} } \right. $$

4 $$ {\text{CBF}}_{50} \left( t \right) = \sum {B_{{\left( {x,y} \right)}} \left( t \right)} $$

where CBF_50_(*t*) is the area with over 50% CBF reduction compared to baseline after the photothrombotic stroke modeling procedure. *t* was set to 15 min in this study. Similarly, we calculated CBF_+_(*t*), which is the area with enhanced CBF post stroke modeling compared with baseline level for further analysis.

### Brain lesion evaluation

In brain lesion evaluation, we performed MRI scanning at 3 h post stroke, which is corresponding to the hyperacute stage in stroke study [23]. The animals were placed in an MRI scanner (Siemens MAGNETOM Trio 3T, Munich, Germany) to evaluate the brain lesion volume in vivo. The scanner was equipped with a dedicated solenoid rat coil (diameter: 60 mm), which was manually tuned and matched. The lesion site was mapped using high-resolution T2-weighted spin-echo imaging. Twenty continuous coronal and twenty continuous transversal slices (thickness: 1 mm) were acquired with the following parameters: field of view, 50 × 50 mm; matrix size, 512 × 512; repetition time, 3000 ms; echo time, 68 ms; number of averages, 2. The total imaging time was about 4 min. Computer-aided planimetric assessment of the lesion volume was performed using ImageJ software [24] blindly. To evaluate the lesion volume, a threshold was applied to MRI images after 3 × 3 pixels Gaussian filtering with the threshold set to 75% maximum intensity of each image. Total lesion volume was subsequently calculated by multiplying the summation of lesion area on each slice with the slice thickness.

The infarct size and NSS were measured at subacute stage (i.e. 24 h) of stroke [23]. The animal behavior performances were evaluated with NSS by 3 experienced examiners independently, who were blind to the experimental grouping. The NSS was presented as mean data (averaged over 3 trials) and graded on a scale from 0 to 18 (normal: 0, maximal deficit score: 18) according to Chen, et al. [25] (Table 1). All rats in both groups were alert during NSS evaluation.

**Table 1:** Neurological severity scores (Modified from Chen et al. [25])

Motor tests	6
Sensory tests	2
Beam balance tests	6
Reflexes absent and abnormal movements	4
Maximum points	18

For each category of assessment, higher score indicates more severe injury

After NSS evaluation, the rats were euthanized and the brains were removed and sectioned coronally (thickness: 3 mm) with brain matrices (Model No. 68710, RWD Life Science Co., Ltd, Shenzhen, China). All brain slices were stained with TTC (2,3,5-triphenyltetrazolium chloride, Sigma-Aldrich Co. LLC, St. Louis, MO) at 37 °C for 10 min in a dark chamber. The infarct volume was quantitated by ImageJ software as the summation of all the slice infarct area multiplied by the slice thickness.

### Statistical analysis

The differences between groups in CBF changes, lesion volumes from MRI analysis, NSS, and infarct volumes from TTC staining were determined by *t* test using MATLAB^®^. Significance level was set at *P* < 0.05. All data were presented as the mean ± SEM.

## Results

### Cerebral blood flow information

**Fig. 2:**
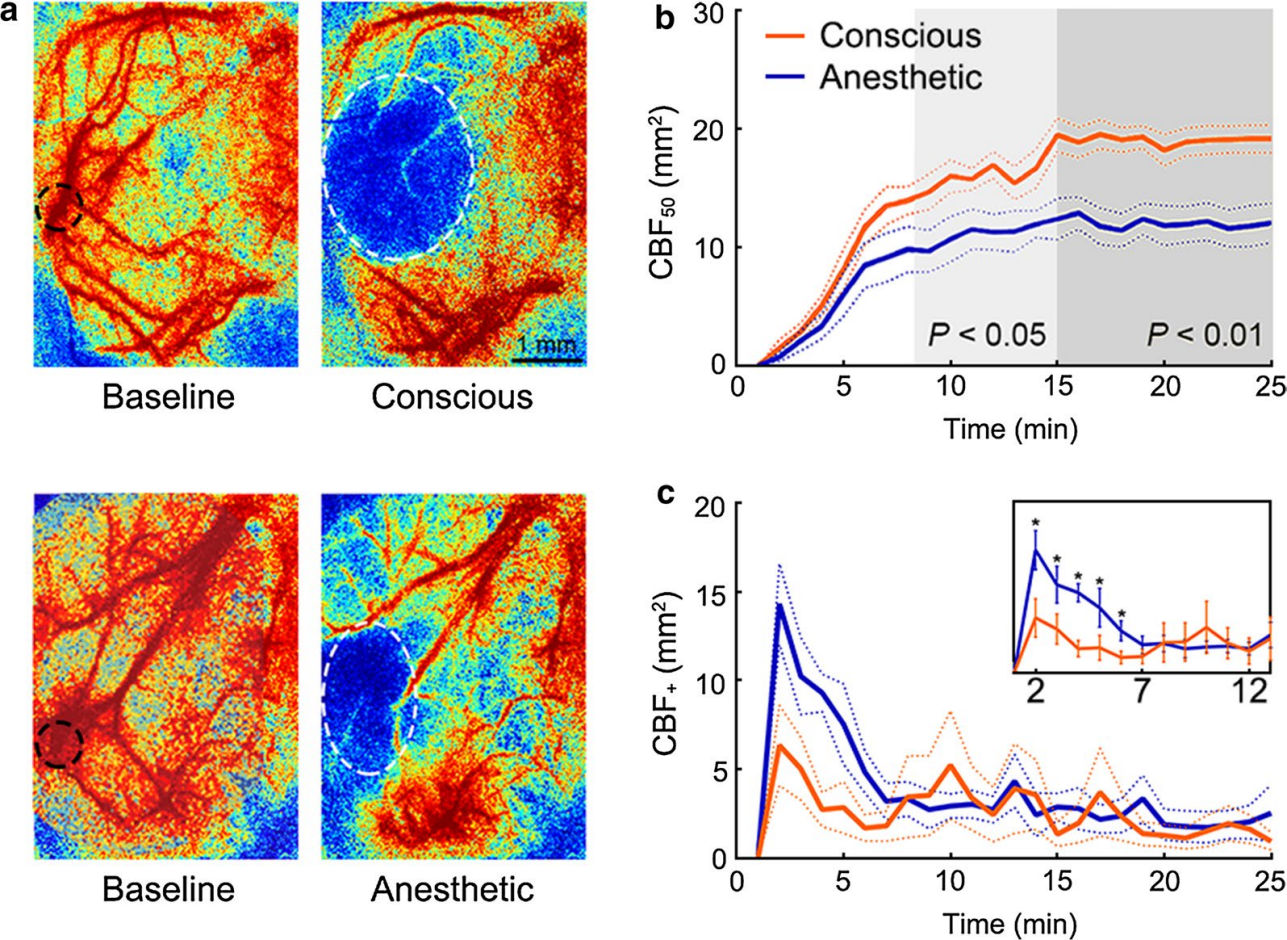
CBF information throughout the modeling of focal cerebral ischemia in the conscious (*n* = *11*) and the anesthetic (*n* = *11*) groups. **a** Representative LSI CBF images of rats from both groups. Images were acquired 15 min after illumination initiation. *White dashed lines* enclose the core CBF_50_, i.e., more than 80% pixels of CBF_50_ are within this area; while *black dashed lines* indicate the focus of the 532 nm light illumination applied in modeling of focal ischemia. *Scale bar* 1 mm. **b** The changes of CBF_50_ throughout the stroke modeling in each group, showing a larger CBF reduction area in the conscious group after 8 min illumination. **c** The changes of CBF_+_ showing a larger area with enhanced CBF in the anesthetic group during modeling. *P < 0.05

The CBF results confirmed that MCAs of all rats in both groups were completely occluded by an intraluminal thrombus formed after 15 min 532 nm laser illumination (regional CBF with over 85% decrease, compared with baseline values obtained before stroke modeling). The changes of CBF_50_ reduction area in both groups were calculated (Fig. 2a). CBF_50_ was compared at each time point by independent samples t-tests. After 8-min illumination, significantly larger CBF_50_ was consistently observed in the conscious group compared with the anesthetized group (*P* < 0.05). And after 15 min illumination, CBF_50_ was 19.5 ± 1.4 mm^2^ in the conscious group, which was significantly larger than that in the anesthetic group (12.4 ± 1.5 mm^2^, *P* < 0.01; Fig. 2b). All CBF changes are expressed as percentages of the baseline values.

The changes of CBF_+_ area along with time in both groups were calculated and shown in Fig. 2c. CBF_+_ at each time point was also compared between two groups by independent samples t-tests. Significantly larger CBF_+_ was observed 1 min after illumination initiation in the anesthetized group compared with the conscious group (*P* < 0.05). After 15-min illumination, CBF_+_ showed a decreasing trend though with no significant between-group difference (*P* > 0.05).

### Brain lesion evaluation

**Fig. 3:**
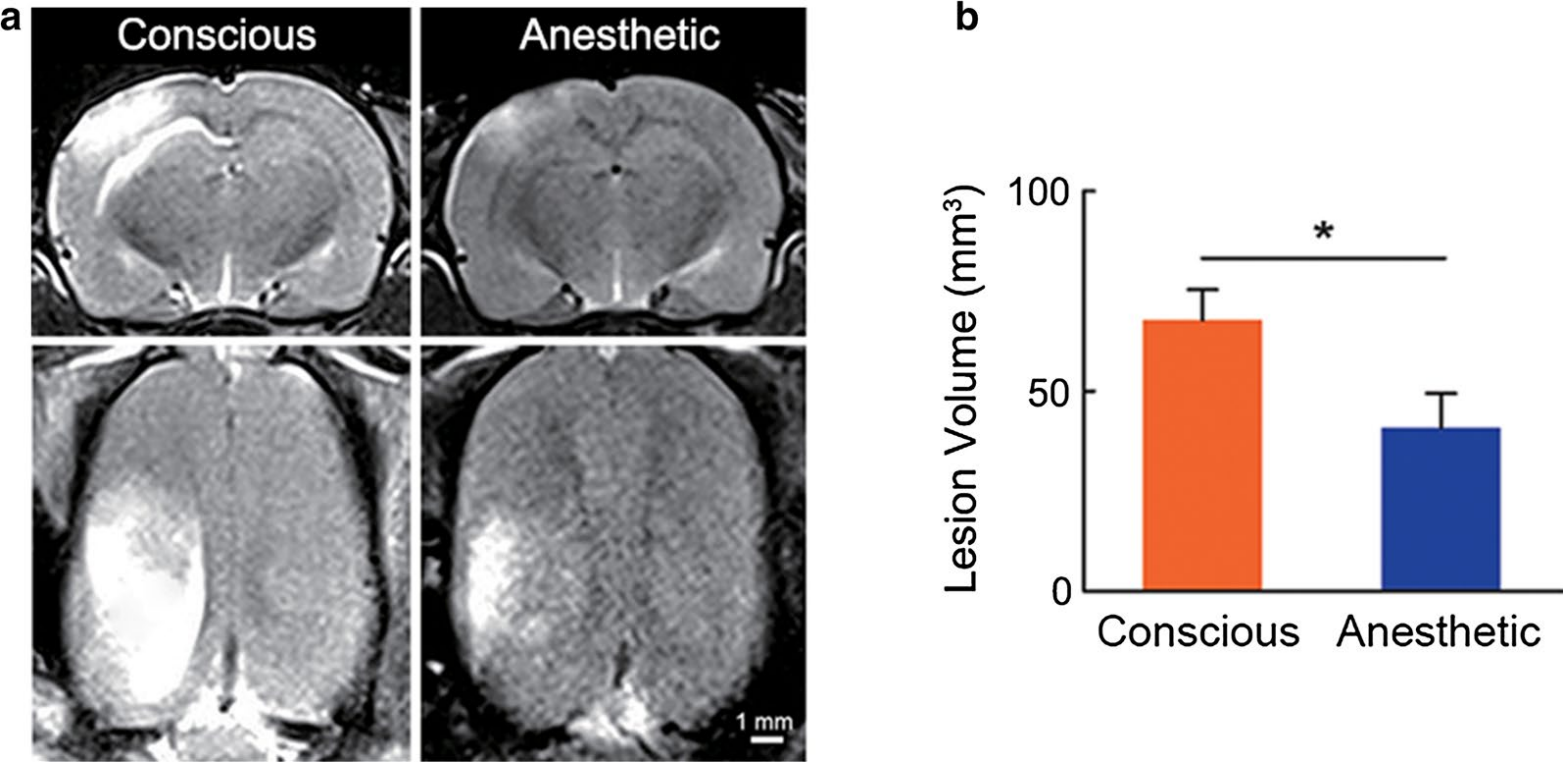
Brain lesion evaluations after photothrombotic stroke modeling in the conscious (*n* = *11*) and the anesthetic (*n* = *11*) groups. **a** Coronal and transversal planes of T2-weighted MRI from representative rats acquired 3 h after occlusion, showing a larger affected area in the conscious rat than that in the anesthetized rat. *Scale bar* 1 mm. **b** Lesion volume identified by T2-weighted MRI in the conscious group was larger than that in the anesthetic group. *P < 0.05

T2-weighted MRI is a sensitive and accurate technology in ischemic cerebral pathology diagnosis. Figure 3a shows the coronal and transversal MRI slices of representative rats from both groups acquired at three hours post stroke. In the conscious group, the ischemic lesion volume was 63.7 ± 11.2 mm^3^, while the anesthetic group showed a much smaller lesion volume as 40.1 ± 6.2 mm^3^ with borderline significance (*P* < 0.05). As shown in Fig. 3b, the intra-group variation was relatively high due to various progression pattern of brain lesion among the animals.

**Fig. 4:**
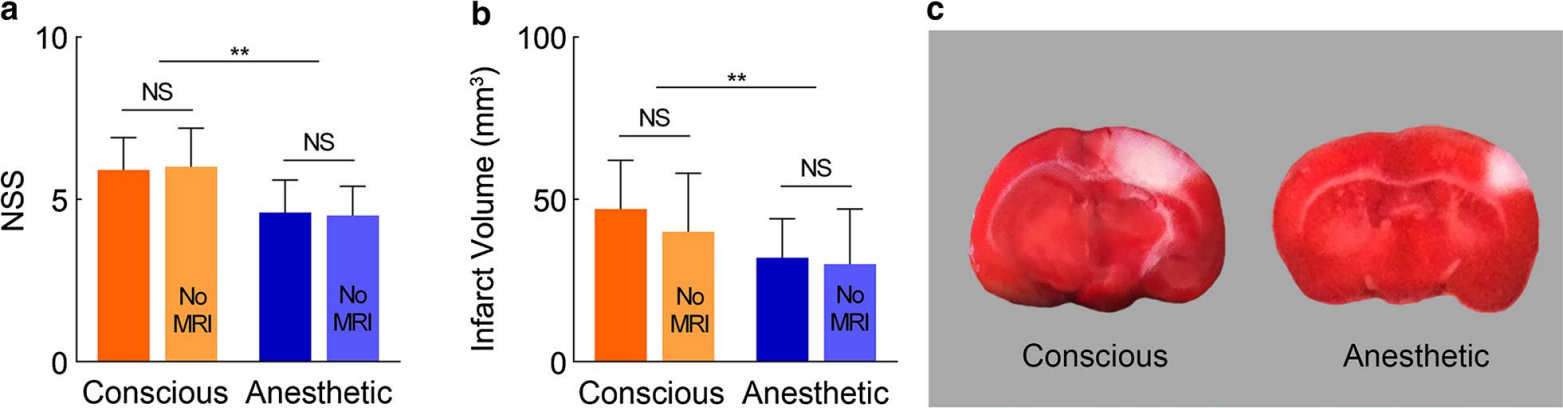
Brain injury evaluations in the conscious and the anesthetic groups. **a**, **b** NSS and infarct volume from TTC staining in the conscious group (*n* = *11*) 24 h post occlusion showed significantly worse score and larger volume than the anesthetic group (*n* = *11*). *P < 0.05; **P < 0.01. An extra experiment was performed with conscious (*n* = *3*) and anesthetic (*n* = *3*) rats going through all the protocol except for MRI scanning (‘No MRI’) showing no significant difference in both NSS and infarct volume compared with the original groups. **c** Representative TTC-stained brain slices showed larger damaged brain areas (*white*) in the conscious group after photothrombotic stroke

In respect of NSS, score points are accumulated for the inability to perform the test or for the lack of a tested reflex; thus, the higher score, the more severe is the injury. In the conscious group, NSS was 5.9 ± 1.0, while NSS in the anesthetic group was 4.6 ± 1.0, which was significantly lower (*P* < 0.01; Fig. 4a). Concordantly, the infarct volume of rats measured 24 h post stroke from the conscious group was 47 ± 15 mm^3^, which was significantly larger than that in the anesthetic group (32 ± 12 mm^3^, *P* < 0.05; Fig. 4b, c). From the above evaluation, the anesthetic group showed a significantly smaller brain lesion than the conscious group, indicating the neuroprotective effects of intraoperative anesthetics.

To address the impact of the anesthetics administered during MRI scanning on the measurement conducted 24 h following stroke, we performed an extra experiment with conscious (*n* = *3*) and anesthetic (*n* = *3*) rats going through all the protocol except for MRI scanning. Both NSS and infarct volume showed no significant difference in comparison with the groups from the original protocol (Fig. 4).

## Discussions

In this study, we compared the CBF changes throughout the photothrombotic stroke modeling of focal cerebral ischemia in conscious and isoflurane anesthetized rats, respectively. NSS, LSI, MRI analysis and TTC staining were applied to evaluate the neurologic deficits and brain lesion after stroke. We found a much smaller CBF reduction area during the surgery in the anesthetic group. Moreover, it was shown that the intraoperative anesthetics provided neuroprotective effects to the ischemic brain injury.

Different imaging techniques have been developed in monitoring the influence of isoflurane on regional CBF during stroke. For instance, a longitudinal MRI study carried out between 6 h and 21 days after ischemia showed that isoflurane altered regional CBF and constrained the deleterious hemodynamic variation in ischemia reperfusion injury [12]. LDF is an in vivo real-time imaging technique, which has been commonly utilized to monitor transient focal CBF throughout surgery or during the induction of ischemia [26, 27]. For example, Bleilevens et al. [27] observed the focal CBF in the ischemic area of isoflurane anesthetized rats by LDF at various time points before and after the onset of ischemia, finding significantly higher values at 50 min after ischemia in comparison with ketamine/xylazine anesthetized rats. Compared with LDF, LSI provides full-field CBF information with high spatial and temporal resolution [28]. Owning to our conscious photothrombotic stroke model, for the first time, we were able to exclude the anesthetic effect during the whole modeling procedure and investigate the intraoperative anesthetic influence on the 2D CBF characteristics. The CBF information acquired during and early after stroke demonstrated that the animal hemodynamics were affected by anesthetics at as early as during the stroke induction and immediately after stroke. Also, our findings about the neuroprotective effect due to intraoperative anesthetics administration during stroke modeling bring additional insights in understanding the translational difficulty in stroke research.

In our present work, the photothrombotic stroke model was adopted. The vascular thrombosis was formed through the photoactivation of the pre-injected Rose Bengal followed by platelet aggregation. However, it was reported that inflammation after experimental stroke could lead to brain edema, blood–brain barrier injury, which would impair the recovery in stroke rats [29]. A number of studies have reported dose-dependent increase in CBF as well as heterogeneous change in CBF distribution with isoflurane administration [30–32]. Also, it was proposed that the neuroprotective effect of isoflurane anesthesia might be caused by constrained deleterious CBF change [12]. Moreover, the CBF reduction in early stage after stroke, both in the ischemia core and in ischemic penumbra, has been shown closely associated with infarction volume [9]. The isoflurane-induced hemodynamic changes might be relevant to cerebral vasodilation and/or collateral circulation enhancement during ischemia, considering the fact that the isoflurane might serve as a potent cerebral vasodilator [33]. For example, the isoflurane-mediated increase of nitric oxide could induce the depolarization of mitochondria in endothelial cells [34]. Moreover, the dilation of arterioles in the ischemic penumbra [35] could be neuroprotective. It is in line with the present study that the CBF reduction area in the isoflurane anesthetized group was more constrained than that in the conscious group, which might contribute to the less brain injury after stroke. Nevertheless, the neuroprotective effect that isoflurane exerts is not solely caused by the hemodynamic changes. Alterations at molecular level, e.g. neurotransmitter concentration and neuronal excitability, could also contribute to the changes in anesthetic property [36–38]. Our work provided a useful tool for the study of early neuroprotective effect in relation to CBF changes during the procedure of stroke modeling. The detailed mechanisms underlying it merit further experimental investigations such as the alteration of neuronal excitability and its association with hemodynamic variations. Moreover, the study on dose-dependent effect of isoflurane in CBF changes could be performed in future work.

## Abbreviations

CBF: cerebral blood flow
LDF: laser Doppler flowmetry
LSI: laser speckle imaging
MCA: middle cerebral artery
MRI: magnetic resonance imaging
NSS: neurological severity score
rCBF: relative CBF
TTC: 2,3,5-triphenyltetrazolium chloride
